# Optimizing control strategies for DC-DC boost converters: Real-time application of an adaptive gain scheduled ISA-PI controller with hybrid state-space and linear parameter-varying modelling

**DOI:** 10.1371/journal.pone.0325969

**Published:** 2025-07-09

**Authors:** Cağfer Yanarateş

**Affiliations:** 1 Department of Electrical and Energy, Kelkit Aydın Doğan Vocational School, Gümüşhane University, Gümüşhane, Turkey; Yanbu Industrial College, EGYPT

## Abstract

This paper introduces an innovative sophisticated control scheme for a DC-DC boost converter (DCBC), employing an adaptive gain scheduled ISA-PI controller. Addressing the inherent non-minimum phase behaviour arising from a right-half plane zero and the complexities associated with nonlinear dynamics during continuous conduction mode (CCM), the proposed adaptive gain scheduled ISA-PI controller incorporates the distinct adjustable parameter within the controller structure. This parameter is instrumental in enhancing the adaptability of the controller to varied operating conditions. The adaptive ISA-PI controller seamlessly integrates the real-time duty cycle value, replacing traditional tuning variables with precision. The dynamic adjustment of this sole controllable parameter is facilitated through a carefully designed look-up table, employing the loop-shaping method. Verification of the proposed control system’s effectiveness is conducted using MATLAB/Simulink, incorporating a comprehensive comparative analysis against single proportional integral (PI) controllers. The assessment centres on evaluating the system’s precision in tracking desired signals and regulating plant process variables with optimal efficiency, minimizing delays and overshoot. Experimental validation is further undertaken using MATLAB/Simulink/Stateflow on a dSPACE Real-time-interface (RTI) 1007 processor, DS2004 High-Speed A/D, and CP4002 Timing and Digital I/O boards. The experimental results confirm the superior performance of the proposed adaptive gain schedule ISA-PI controller, which has a unique configurable parameter. This controller demonstrated a twofold improvement in tracking speed and significantly improved disturbance rejection, confirming its effectiveness.

## 1. Introduction

DC-DC boost converters (DCBCs) are critical components in power electronics, with applications ranging from renewable energy systems to small electronic devices [1–3]. Their widespread use underlines their strategic importance in the quest for efficient and durable power sources [4,5]. As a type of DC-DC switch mode power supply, DCBCs offer several advantages, including being more compact, lighter, less noisy and highly efficient, making them indispensable in modern power electronic systems [6–8].

Despite their benefits, the management of DCBCs presents significant challenges that require innovative solutions to optimise their operational efficiency [9,10]. Key issues arise from the presence of a right half-plane zero (RHPZ) in their transfer functions, leading to non-minimum phase (NMP) behaviour [11–13]. This NMP characteristic complicates control design and often leads to unexpected problems such as undershoot in response to control inputs [14,15]. In addition, managing the NMP dynamics of the RHPZ further complicates the stability and responsiveness of the control system, making the efficient operation of DCBCs in power electronics a complex task [16–18].

NMP systems, particularly those with RHPZ, present unique control design challenges [19]. The NMP behaviour results from the phase lag caused by the transfer of stored energy from the turn-on period to the load during the turn-off period, which limits the control bandwidth achievable by feedback control [20]. This problem is related to the presence of RHPZ in the transfer function from control to output voltage, which complicates the control effort [21]. In addition, dynamic shifts in the location of the RHPZ, driven by changes in converter parameters such as voltage gain and load resistance, exacerbate the problem [22]. For example, a reduction in input voltage or load resistance moves the RHPZ closer to the origin, destabilizing the closed-loop system [23].

These challenges highlight the importance of tailored control solutions for the effective and reliable operation of DCBCs [24]. Adaptive control strategies are required to cope with the dynamic nature of NMP systems in power electronics [25,26]. In order to improve the control performance, this study introduces an adaptive mechanism that enhances the conventional Proportional-Integral (PI) controller and develops it into an ISA-PI controller [27,28]. This modification aims to achieve two key objectives: improved disturbance rejection and improved reference tracking, ultimately addressing the complexities associated with NMP behaviour in DCBCs.

The ISA-PI controller introduces an additional tuning parameter, $d^{m}$, which allows independent control of the effect of the reference signal on the proportional action, significantly improving the controller’s responsiveness in reference tracking. To further increase the controller’s adaptability, the real-time duty cycle is integrated into the control system, replacing the conventional $d^{m}$ parameter with a dynamically varying value. This integration is supported by a carefully designed look-up table, developed using the loop-shaping method to facilitate real-time adaptation [25].

The advanced control strategy of the ISA-PI controller, combined with the adaptive integration of the duty cycle through the custom look-up table, maximises both noise rejection and reference tracking performance. This approach highlights the effectiveness of adaptive control techniques in the context of DCBCs, with the loop-shaping method providing a foundation of technical precision and rigour in the controller design [22].

The rest of the paper is structured as follows. Section 2 delves into the performance evaluation of the proposed adaptive gain scheduled ISA-PI controller, highlighting its advantages and key considerations, with a focus on how adaptive control can overcome conventional limitations. Section 3 outlines the design of the proposed adaptive gain scheduled ISA-PI controller, detailing its components and mechanisms. Section 4 introduces linear parameter-varying (LPV) systems and their application in control system design. Section 5 presents the results and discussion, covering both simulation and experimental results. Finally, Section 6 concludes the study by summarizing the findings and providing insights into possible future work.

## 2. Performance evaluation of the proposed adaptive gain scheduled ISA-PI controller: Advantages and considerations

Before exploring the proposed adaptive ISA-PI controller, it is important to first acknowledge both the strengths and limitations of traditional PI controllers [27,29]. Widely used for their simplicity, versatility and ease of implementation, PI controllers are effective in many control applications due to their balance between fast response and steady-state accuracy through proportional and integral action [30–32]. However, despite these advantages, PI controllers face significant challenges, particularly in switch mode power supply (SMPS) systems such as DCBCs. In these scenarios, PI controllers often struggle with inadequate transient response, sensitivity to parameter variations and difficulties in managing complex system dynamics. The non-linear and time-varying nature of SMPS applications exacerbates these problems, making conventional PI controllers less effective [14]. For example, right-hand half-plane zeros in transfer functions introduce control challenges such as undershoot, which further degrade system performance. Consequently, there is a need for more advanced control strategies, particularly in DCBCs where the limitations of the traditional PI controller become apparent. Table 1 outlines the advantages and key considerations of the proposed adaptive gain scheduled ISA-PI controller, which provides a solution to these challenges.

**Table 1:** Advantages and considerations of the proposed adaptive gain scheduled ISA-PI controller.

Parameters/Aspects	Advantages	Considerations
**Adaptability**	Improved adaptability to varying system conditions.	Requires careful tuning for optimal performance.
**Reference Tracking**	Enhanced reference tracking response.	Sensitivity to extreme variations in reference signals.
**Disturbance Rejection**	Effective disturbance rejection capabilities.	Performance may be impacted in the presence of severe and frequent disturbances.
**Complexity**	Strikes a balance between performance and simplicity.	May introduce complexity, depending on the tuning requirements.
**Robustness**	Improved robustness against uncertainties and variations.	The extent of robustness may be influenced by the tuning process.
**Stability**	Enhanced stability in dynamic operating conditions.	Sensitivity to improper tuning and modelling inaccuracies.
**Performance** **Trade-off**	Offers a favourable trade-off between performance and complexity.	Balancing competing objectives may require iterative tuning.
**Switch Mode Power Supplies**	Addresses inadequacies of traditional controllers in switch mode power supplies.	Sensitivity to specific characteristics of different power supply configurations

Although more advanced control strategies, such as model-based approaches and predictive control, can outperform simpler methods under ideal conditions, their complexity and sensitivity to parameter uncertainties pose challenges for real-world implementation [33,34]. These techniques often require precise modelling and complex tuning, limiting their practicality in many applications [35]. In contrast, adaptive control offers a compelling solution to overcome the limitations of traditional controllers [36]. It addresses the dynamic nature of systems, operational variations and uncertainties by continuously adapting and optimizing performance [37]. Adaptive control techniques improve robustness, tracking accuracy and disturbance rejection, making them highly effective in managing evolving conditions.

## 3. Proposed adaptive gain scheduled ISA-PI controller design

**Fig 1:**
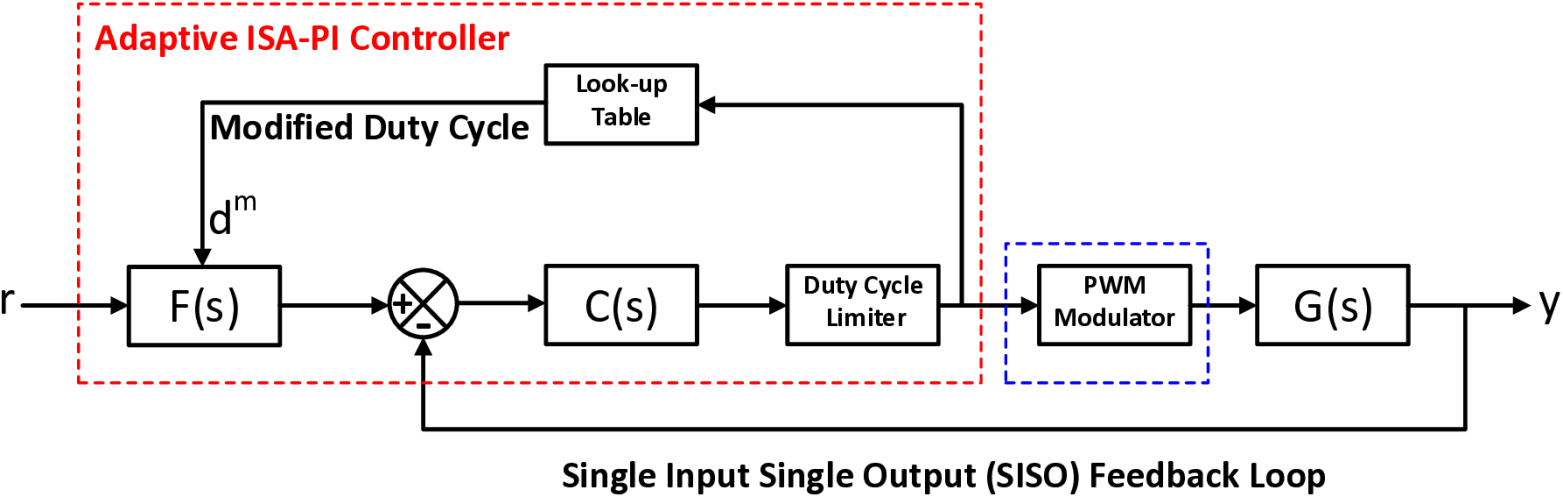
Overview of the adaptive gain scheduled ISA-PI control system.

The transition to an Adaptive Gain ISA-PI controller offers a strategic solution to overcome limitations associated with conventional PI controllers, especially in the context of switch mode power supply applications such as the DCBC Fig 1. providing an overview of the entire proposed adaptive Gain Scheduled ISA-PI control system.

**Fig 2:**
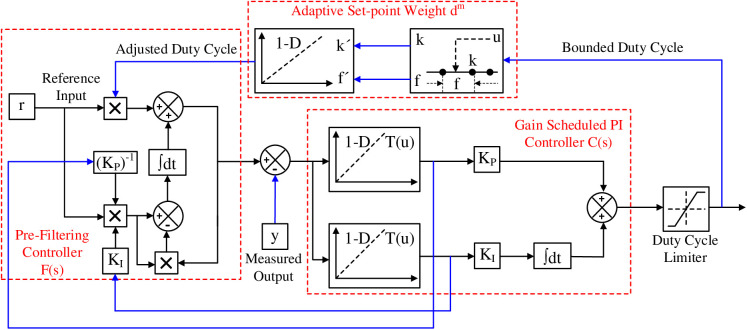
Internal components of the adaptive gain ISA-PI control scheme.

In Fig 2, the proposed control scheme’s internal components and mechanisms for dynamically adjusting controller gains are highlighted. The blue path illustrates the feedback loop, while the black path represents the feedforward path.

**Fig 3:**
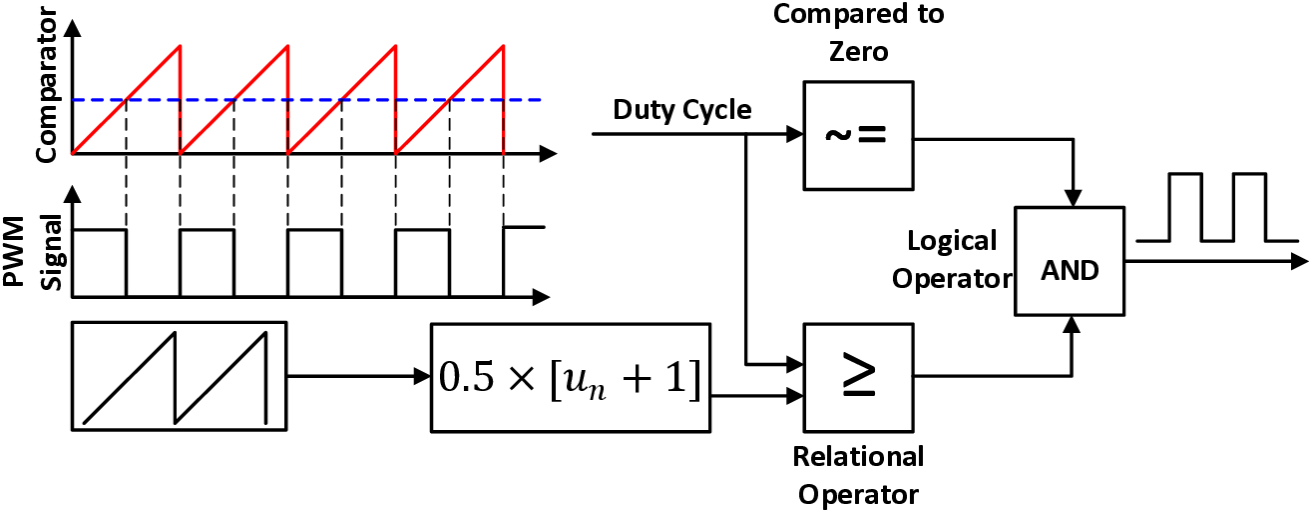
Operational aspects of PWM generation in power transfer control.

Fig 3 illustrates the operational aspects of PWM generation which controls power transfer from one electrical component to another by quickly switching between full power transfer and no power transfer.

Considering the aforementioned challenges, the proposed adaptive ISA-PI controller emerges as a practical solution. By introducing an independent tuning parameter to the conventional PI controller, the ISA-PI design significantly improves its adaptability to varying reference signals and disturbances. The numerical value of the set-point weight, $d^{m}$, is constrained between 0 and 1. This adaptive feature provides a balanced trade-off between performance and complexity, allowing the controller to overcome the limitations of traditional PI controllers, particularly in demanding applications such as switching power supplies. The adaptive ISA-PI controller aims to improve stability, responsiveness and robustness in dynamic systems, effectively filling the gaps left by conventional controllers while avoiding the complications associated with more complex control strategies.

### 3.1. Boost converter power stage

The state-space modelling of the boost converter employs a dynamic (AC small signal) methodology, using a transfer function model [38,39]. This approach involves applying the zero-conditioned Laplace transform to the state and output equations within the state-space representation of the boost converter. The following design requirements are used to derive the nominal values: the maximum ripple permitted in the capacitor voltage is 20% of the average inductor current at maximum load, and the maximum ripple permitted in the inductor current is ± 2%. Constrained budgets, the use of pre-existing components, ageing materials, environmental factors that can impact performance and reliability, variances in manufacturing processes, component tolerances, and the complexity of real-world operating conditions are some of the reasons why designing ideal systems is frequently impractical in practice. As a result, it is critical to first recognize and resolve these constraints. These issues and challenges in applications can be mitigated by developing a robust and sophisticated controller which constitutes one of the main and prominent subjects of this study. Table 2 presents parameter calculations of the ideal model operating condition in continuous conduction mode (CCM).

**Table 2:** Ideal model parameters derived from design requirements.

Parameter	Equation	Nominal Value
SW	$\frac{V_{out}}{V_{dc}}=\frac{1}{\left(1-D\right)}$	Duty ratio of 0.5
Average Inductor Current ($I_{L}$)	$\frac{{V_{out}}^{2}}{RV_{dc}}$	4A
Inductor Ripple Current ($\mathrm{\backslash Updelta}I_{L}$)	$0.2\times I_{L}$	0.8A
L	$\frac{V_{in}D}{f_{sw}\mathrm{\backslash Updelta}I_{L}}$	0.9375 mH
Capacitor Ripple Voltage ($\mathrm{\backslash Updelta}V_{C}$)	$0.04\times V_{out}$	2.4V
$C_{out}$	$\frac{V_{in}D}{Rf_{sw}\mathrm{\backslash Updelta}V_{C}(1-D)}$	$\begin{array}{c} ≅20.833\;\mu \text{F} \end{array}$
$RC_{out}$	$R\times C_{out}$	$\begin{array}{c} ≅6.25\times 10^{-4}\;s \end{array}$

In this context, Table 3 provides a detailed overview of the critical components integrated into the proposed DCBC design, including essential elements such as diodes, active switches, capacitors, and inductors.

**Table 3:** Overview of key components in the proposed DCBC design.

Component	Symbol	Value	Impact on Controller Performance
Inductor	L	2 mH	Rapid response to input changes
Switch (Duty Ratio)	d	0.5	Affects settling time and transient behavior.
Switching Frequency	$f_{sw}$	20 kHz	Impacts system performance at high frequencies by influencing rapid response, transient behavior, output quality, and overall efficiency.
Capacitor	$C_{out}$	440 μF	Affects output voltage quality.
Input Voltage	$V_{dc}$	30 V	Determines initial system energy
Output Voltage	$V_{out}$	60 V	Specifies target voltage level.
Load Resistance	R	30 Ω	Alters response to load changes.
Time Constant	$RC_{out}$	0.0132 s	Influences overall system dynamics.

The designed output capacitance is 440 µF, while the nominal value is approximately 20.833 µF. This difference results in approximately a 95.27% reduction in output voltage ripple, a 2015% increase in rise time, and a decrease in overshoot ranging between 50% and 70%. Similarly, the designed inductance value is 2 mH, whereas the nominal value is 0.9375 mH. This discrepancy leads to an approximate 122% increase in rise time and around a 55% reduction in overshoot, significantly influencing both the step and frequency response characteristics. Additionally, the time constant of the designed DCBC is 0.0132 s, while the nominal value is approximately 6.25 × 10^-4^ s. This substantial difference strongly influences the overall system dynamics, impacting both transient and steady-state behavior. The proposed control system, as previously discussed in detail, is designed to ensure the system operates efficiently even under non-ideal conditions.

The circuit analysis of the DCBC in CCM is presented in Table 4, encompassing detailed examinations during both the time intervals when the active switch MOSFET is in the ON and OFF states.

**Table 4:** Circuit analysis of the DCBC in continuous conduction mode.

[\textbf{State}1:SWON,DOFF] (Time interval: 0 < t < dT_s_)	[\textbf{State}2:SWOFF,DON] (Time interval: dT_s_ < t < T_s_)
• The controllable switch (MOSFET) is conducting. • Inductor stores energy from the input source. • Capacitor supplies current to the load. $V_{dc}=L\frac{di_{L}}{dt};i_{C}=C\frac{dV_{C}}{dt};V_{C}=-i_{C}R;V_{out}=V_{C}$	• The controllable switch (MOSFET) is in the non-conduction state. • Inductor releases energy to the load via the diode. • Capacitor continues to smooth the output voltage. $V_{dc}=L\frac{di_{L}}{dt}+V_{out};i_{C}=C\frac{dV_{C}}{dt};i_{L}=i_{C}+\frac{V_{out}}{R};V_{out}=V_{C}$

**Fig 4:**
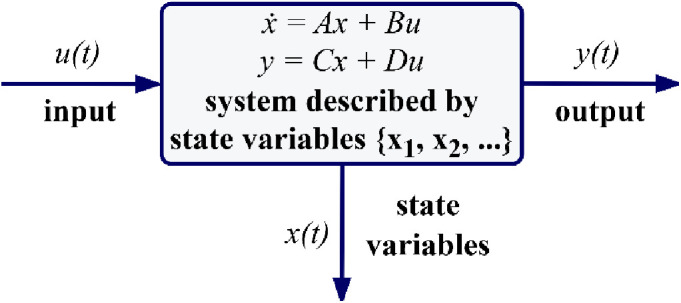
The generic state space representation.

The equations representing the state-space average model of the DCBC for both ON and OFF states of the MOSFET and additionally derivation of the transfer function facilitated by using these equations in conjunction with small-signal AC model are provided in Table 4. Transfer function estimation is facilitated by utilizing these equations, in conjunction with small-signal AC analysis. The most comprehensive and generic state-space representation of a system with p inputs, q outputs, and n state variables is shown in Fig 4.

where A(.), B(.), C(.), and D(.) are state matrix with dim[A(.)]=n×n, input matrix with dim[B(.)]=n×p, output matrix with dim[C(.)]=q×n and feedforward matrix with dim[D(.)]=q×p, respectively. x(.), y(.), and u(.) are state vector with $x(tin{\mathbb{R}}^{n}$, output vector with $y(t)\in {\mathbb{R}}^{q}$, control vector with $u(tin{\mathbb{R}}^{p}$, respectively. In electric circuits, the number of state variables is frequently, though not invariably, identical to the number of energy storage devices present, such as capacitors and inductors. Consequently, the current flowing through the inductor ($i_{L}$) and the voltage across the capacitor ($V_{C}$) are identified as state variables in this context, given that the proposed boost converter comprises a single inductor and a single capacitor. The state vector of the system is expressed as follows:


$$x=\left[\begin{array}{c} x_{1}\\ x_{2} \end{array}\right]=\left[\begin{array}{c} i_{L}\\ V_{C} \end{array}\right]$$ (1)

The state-space equations of the proposed DCBC are presented as follows:


$$\dot{x}=\frac{dx}{dt}=\left[\begin{array}{c} \frac{di_{L}}{dt}\\ \frac{dV_{C}}{dt} \end{array}\right]=Ax+BV_{dc};y=V_{out}=Cx$$ (2)

The transfer function of the converter under consideration has been obtained through the utilization of data from Tables 4 and 5, as follows:

**Table 5:** State-space average model and transfer function derivation of the DCBC.

State-space Average Model	Small-signal AC Model
$\begin{array}{c} \left[\text{\textbackslash{}textbf\{State}\}\;1:SWON,DOFF\right] \end{array}$ (Time interval: 0 < t < dT_s_) $\dot{x}=A_{1}x+B_{1}V_{dc}$ $y_{1}=C_{1}x$ $A_{1}=[\begin{array}{cc} 0 & 0\\ 0 & -1/RC_{out} \end{array}];B_{1}=\left[\begin{array}{c} 1/L\\ 0 \end{array}\right];C_{1}=\left[\begin{array}{cc} 0 & 1 \end{array}\right]$ $\left[\begin{array}{c} {\dot{x}}_{1}\\ {\dot{x}}_{2} \end{array}\right]=[\begin{array}{cc} 0 & 0\\ 0 & -1/RC_{out} \end{array}]\left[\begin{array}{c} x_{1}\\ x_{2} \end{array}\right]+\left[\begin{array}{c} 1/L\\ 0 \end{array}\right]V_{dc}$ $V_{out}=\left[\begin{array}{cc} 0 & 1 \end{array}\right]\left[\begin{array}{c} x_{1}\\ x_{2} \end{array}\right]$	Description of notations d≡dutyratio=0.5 $d^{\prime }\equiv 1-d=0.5$ $T_{sw}\equiv \mathrm{switching}\mathrm{period}=5\times 10^{-5}s$ Basic Averaged Model $\dot{x}=Ax+BV_{dc}$ $A=dA_{1}+d^{\prime }A_{2}=[\begin{array}{cc} 0 & -d^{\prime }/L\\ d^{\prime }/C_{out} & -1/RC_{out} \end{array}]$ $B=dB_{1}+d^{\prime }B_{2}=\left[\begin{array}{c} 1/L\\ 0 \end{array}\right]$ $C=dC_{1}+d^{\prime }C_{2}=\left[\begin{array}{cc} 0 & 1 \end{array}\right]$ Perturbation $d=D+\hat{d}$ $x=X+\hat{x}$ $y=Y+\hat{y}$ $v_{dc}=V_{dc}+{\hat{v}}_{dc}$ Steady-state DC Model $\dot{x}=AX+BV_{dc}=0\Rightarrow X=-A^{-1}BV_{dc}$ $X=\left[\begin{array}{c} V_{dc}/d^{\prime 2}R\\ V_{dc}/d^{\prime } \end{array}\right]$ AC Small Signal Model $\hat{\dot{x}}=A\hat{x}+B{\hat{v}}_{dc}+[\left(A_{1}-A_{2}\right)X+\left(B_{1}-B_{2}\right)V_{dc}]\hat{d\;}$ Transfer Function Derivation $\frac{V_{out}(s)}{d(s)}=\frac{-sLV_{dc}+RV_{dc}d^{\prime 2}}{s^{2}(LRC_{out}d^{\prime 2})+s(Ld^{\prime 2})+Rd^{\prime 4}}$
$\begin{array}{c} \left[\text{\textbackslash{}textbf\{State}\}\;2:SWOFF,DON\right] \end{array}$ (Time interval: dT_s_ < t < T_s_) $\dot{x}=A_{2}x+B_{2}V_{dc}$ $y_{2}=C_{2}x$ $A_{2}=[\begin{array}{cc} 0 & -1/L\\ 1/C_{out} & -1/RC_{out} \end{array}];B_{2}=\left[\begin{array}{c} 1/L\\ 0 \end{array}\right];C_{2}=\left[\begin{array}{cc} 0 & 1 \end{array}\right]$ $\left[\begin{array}{c} {\dot{x}}_{1}\\ {\dot{x}}_{2} \end{array}\right]=[\begin{array}{cc} 0 & -1/L\\ 1/C_{out} & -1/RC_{out} \end{array}]\left[\begin{array}{c} x_{1}\\ x_{2} \end{array}\right]+\left[\begin{array}{c} 1/L\\ 0 \end{array}\right]V_{dc}$ $V_{out}=\left[\begin{array}{cc} 0 & 1 \end{array}\right]\left[\begin{array}{c} x_{1}\\ x_{2} \end{array}\right]$	


$$G\left(s\right)=\frac{-0.06s+225}{6.6\times 10^{-6}s^{2}+0.0005s+1.875}$$ (3)

### 3.2. PI and prefiltering controllers design

**Fig 5:**
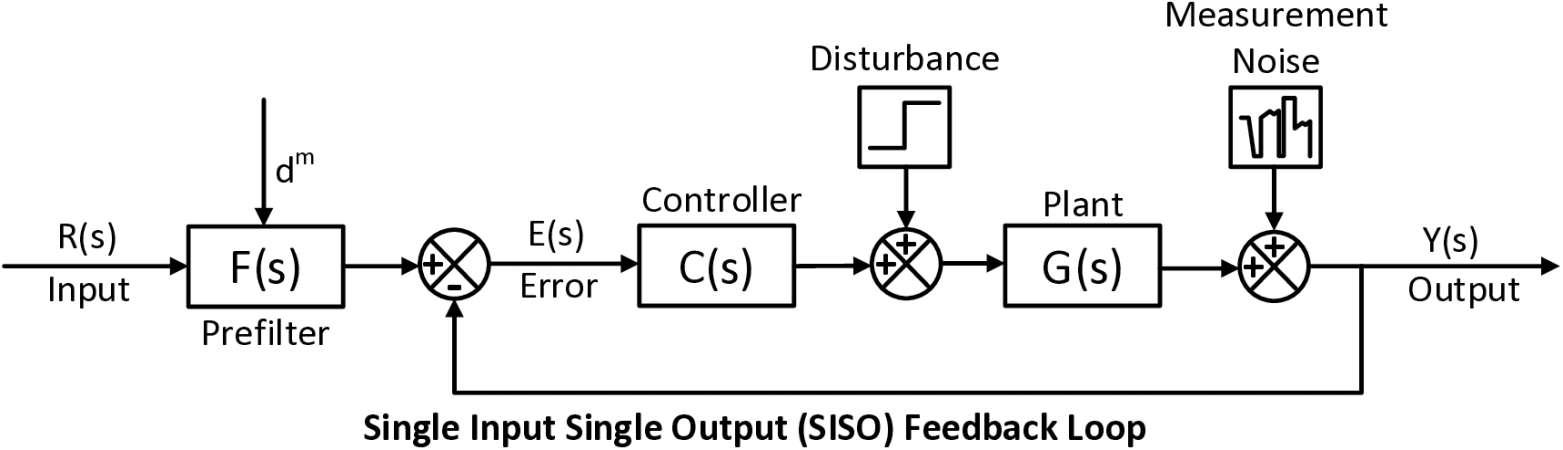
System configuration: prefilter, controller, and plant structure.

The system configuration can be conceptualized as comprising a prefilter, controller, and plant, structured accordingly, as illustrated in Fig 5. Under the premise of negligible input and output disturbances within the system, along with the constancy of set point weight parameters (designated as $d^{m}$).

The transfer function of the closed-loop system T(s) is as follows:


$$T\left(s\right)=\frac{C\left(s\right)G\left(s\right)}{1+C\left(s\right)G\left(s\right)}F(s)$$ (4)

The transfer function of the PI controller, designed by incorporating loop-shaping techniques considering the characteristics of both time and frequency domain analyses, is provided as follows:


$$C\left(s\right)=K_{P}+\frac{K_{I}}{s}=0.0014+\frac{0.151}{s}$$ (5)

where $K_{P}$ and $K_{I}$ are the proportional and integral gains, respectively. The design process utilized specific criteria to ensure optimal performance, including a crossover frequency within the range of 1/10th to 1/8th of the switching frequency, a phase margin exceeding $45^{\circ }$, a gain margin greater than 10dB, and a slope of the gain curve at the crossover frequency approximating −20dB/decade. Additionally, the design aimed to maintain a steady-state error of less than 2% for step inputs. Through the application of root locus and frequency domain analysis, the $K_{P}$ and $K_{I}$ gains were iteratively adjusted to enhance system performance. The implementation of loop shaping in simulation further refined the controller design, ensuring that the desired frequency response was achieved. This systematic approach effectively identifies the gains that yield a robust controller, ultimately resulting in improved performance for the DC-DC boost converter application.

The prefilter transfer function is defined as:


$$F\left(s\right)=\frac{K_{P}d^{m}s+K_{I}}{K_{P}s+K_{I}}=\frac{0.0014\times d^{m}s+0.151}{0.0014s+0.151}$$ (6)

The closed-loop transfer function of the system is reformulated utilizing the provided information as:


$$T\left(s\right)=\frac{\frac{0.0014s+0.151}{s}\times \frac{-0.06s+225}{6.6\times 10^{-6}s^{2}+0.0005s+1.875}}{1+\frac{0.0014s+0.151}{s}\times \frac{-0.06s+225}{6.6\times 10^{-6}s^{2}+0.0005s+1.875}}\times \frac{0.0014\times d^{m}s+0.151}{0.0014s+0.151}$$ (7)

$$T\left(s\right)=\frac{\left(-0.06s+225\right)\left(0.0014d^{m}s+0.151\right)}{6.6\times 10^{-6}s^{3}+5.81\times 10^{-4}s^{2}+2.1839s+33.975}$$ (8)

$$T(s)=\frac{-8.4\times 10^{-5}d^{m}s^{2}+\left(0.315d^{m}-9.06\times 10^{-3}\right)s+33.975}{6.6\times 10^{-6}s^{3}+5.81\times 10^{-4}s^{2}+2.1839s+33.975}$$ (9)

**Fig 6:**
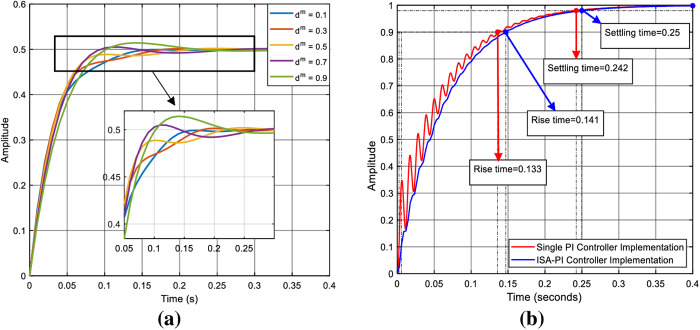
The independent impact of the set point and the assessment of the proposed control mechanism a) impact of set point weight on system response, b) comparison of single PI and ISA-PI control techniques at fixed set-point value of $\begin{array}{c} d^{m}=0.1 \end{array}$.

The impact of changes in the set point weight on the system, under conditions where input-output disturbances, uncertainties, and parameter variations are not considered, and the system response in the presence of noise and disturbance by using single PI and ISA-PI controller with a $d^{m}$ value of 0.1 are depicted in the provided Fig 6a, b, respectively.

The objective is to illustrate that despite the converter being designed with nominal values, variations in the set point weight still influence the performance of the system in the absence of the PI controller. Additionally, the reference tracking response achieved with an ISA-PI controller exhibits smoother waveforms with reduced disturbances.

## 4. Linear parameter-varying systems

LPV models are essential tools in control engineering, allowing the approximation of nonlinear system behaviour and facilitating the application of linear design techniques [40,41]. These models account for variations in system dynamics resulting from changing parameters, providing a linear representation that supports the analysis of key attributes such as stability, controllability and observability [42]. LPV models can be adapted to changing operating conditions by modifying time dependencies or parameters. By simulating LPV models, control strategies can be improved by providing insight into system performance under different settings. Ultimately, these models provide a flexible framework that combines the ability to handle the complexity of dynamic real-world systems with the analytical tractability inherent in linear approaches.

### 4.1. LPV approximation of boost converter model

**Fig 7:**
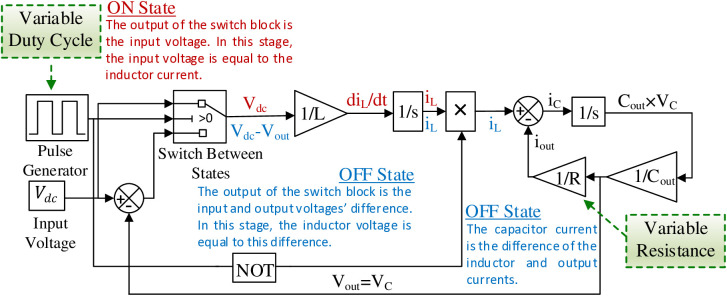
The differential equations model of the DCBC for LPV approximation.

Obtaining a mathematically accurate model that closely resembles the real system is essential for effective control design. In this study, hybrid modelling is applied, integrating state-space and LPV approaches. The LPV concept, derived from gain scheduling for nonlinear systems, enables the use of established linear control design techniques, enhancing adaptability to varying conditions. Meanwhile, state-space representation offers a compact framework for analyzing multi-input, multi-output systems, facilitating state-feedback controller design. This hybrid approach combines the strengths of both methodologies, improving robustness and overall performance metrics, such as stability and response time. Additionally, determining the parameters of the lookup tables and the gains of the PI controller, as well as the adaptive parameters in the system, is effectively achieved through this approach. The differential equations model of the DCBC, as used in LPV approximation through the implementation of varying duty cycles and resistive loads, is presented in Fig 7.

This application uses a state-space variant of an LPV model for the suggested DCBC given by parameter-dependent coefficients, which can be formally expressed as follows:


dx(t)=A(d)x(t)+B(d)u(t)

y(t)=C(d)x(t) (10)

In continuous-time systems, the state derivative vector $\dot{x}$ is represented by dx(t), where u(t) stands for the input voltage $V_{dc}$, y(t) for the output voltage $V_{out}$, and x(t) for the model states inductor current $i_{L}$ and capacitor voltage $V_{C}$. A(d), B(d), and C(d) are state-space matrices parameterized by the scheduling parameter vector d. The scheduling space over which the LPV model is characterised is defined by the functions d=d(t) that are measurable from $V_{dc}$, $i_{L}$, and $V_{C}$.

The affine form of the LPV model can be expanded to include offsets in the dx, x, u, and y variables. This can be stated mathematically as follows:


$$dx(t)=A(d)x(t)+B(d)u(t)+(\overset{―}{dx}(d)-A(d)\overset{―}{x}(d)-B(d)\overset{―}{u}(d))$$

$$y\left(t\right)=C\left(d\right)x\left(t\right)+(\overset{―}{y}\left(d\right)-C\left(d\right)\overset{―}{x}\left(d\right))$$ (11)

The values of $\overset{―}{dx}\left(d\right)$, $\overset{―}{x}\left(d\right)$, $\overset{―}{u}\left(d\right))$, and $\overset{―}{y}\left(d\right)$ indicate the offsets in the values of dx(t), x(t), u(t) and y(t) at a given parameter value d=d(t).

Upon scrutinizing the matrices representing the state, input, and output of the system, along with the corresponding vectors given in Table 4, it becomes apparent that the model is directly susceptible to fluctuations in the input voltage ($V_{dc}$) and the load resistance (R). Specifically, variations in the input voltage induce changes in the duty cycle ($d^{\prime }$) of the permanent regime, while alterations in the load resistance are consequential to load variations. Consequently, if the duty cycle is contingent upon the input voltage, and the load is contingent upon power, it can be posited that the system described by Equation (9) exhibits Linearity Parameter Varying (LPV) characteristics. Accordingly, the system can be formally expressed as:


$$A(d^{\prime },R)=[\begin{array}{cc} 0 & -d^{\prime }/L\\ d^{\prime }/C_{out} & -1/RC_{out} \end{array}]$$ (12)

In the presence of input voltage variations resulted in change in the duty cycle, denoted as $d^{\prime }\in [\underset{―}{d^{\prime }}\overset{―}{d^{\prime }}]$, and load resistance fluctuations, denoted as $R\in [\underset{―}{R}\overset{―}{R}]$, the system exhibits variability characterized by uncertain parameters. In this study, ten specific points for trimming and linearization have been meticulously selected and applied to enhance the accuracy of the model under consideration. Thus, the proposed system is encompassed within a polytope formed by ten local points as:


$$\left[A\left(d^{\prime },R\right)\right|B\left(d^{\prime },R\right)|C(d^{\prime },R)]\in C_{set}\left\{\left[A_{0},B_{0},C_{0}\right],\left[A_{1},B_{1},C_{1}\right],\left[A_{2},B_{2},C_{2}\right],\ldots ,[A_{9},B_{9},C_{9}]\right\}$$ (13)

where $C_{set}\{.\}$ represents the convex hull of the polytope, and $\left[A_{j},B_{j},C_{j}\right]$ denote the vertices comprising the polytopic structure. Thus, the system matrix A can be reformulated as follows:


$$A\left(d^{\prime },R\right)=A_{0}+\sum_{i=1}^{n=9}d_{i}^{\prime }A_{i}+\sum_{i=1}^{n=9}R_{i}A_{i}$$ (14)

$$A\left(d^{\prime },R\right)=A_{0}+d_{1}^{\prime }A_{1}+R_{1}A_{1}+d_{2}^{\prime }A_{2}+R_{2}A_{2}+\ldots +d_{9}^{\prime }A_{9}+R_{9}A_{9}$$ (15)

The linear system array is obtained through the process of linearization across a specified set of operating points, where the offsets are aligned. This set of operating points encompasses variations in duty cycles, specifically involving 10 distinct duty cycle values varying between 10%and80% shown as below.


$$d^{\prime }=[0.1,0.1778,0.2556,0.3333,0.4111,0.4889,0.5667,0.6444,0.7272,0.8]$$ (16)

A vector is formed by choosing 10 distinct values within the range of 20–40 ohms to represent approximately ∓33% variations in load, as illustrated below.


R=[20,22.2222,24.4444,26.6667,28.8889,31.1111,33.3333,35.5556,37.7778,40] (17)

**Fig 8:**
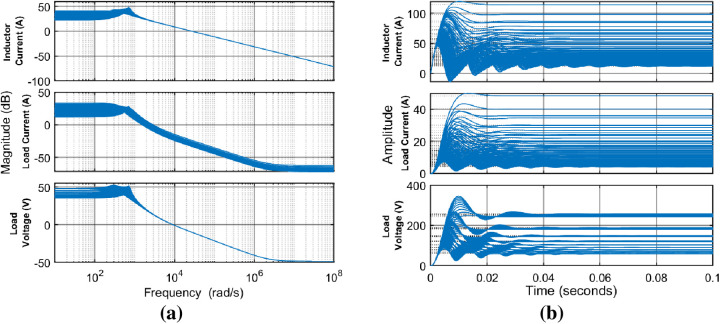
Thorough visualization of linear system array with 10 distinct points withing the range of 20-40 Ω representing $\begin{array}{c} \mp 33\% \end{array}$ load variation a) frequency response, b) step response.

The linear system array is visualized comprehensively, presenting both time-domain step response plot and frequency spectra in the accompanying Fig 8a, b, respectively.

## 5. Results and discussions

**Fig 9:**
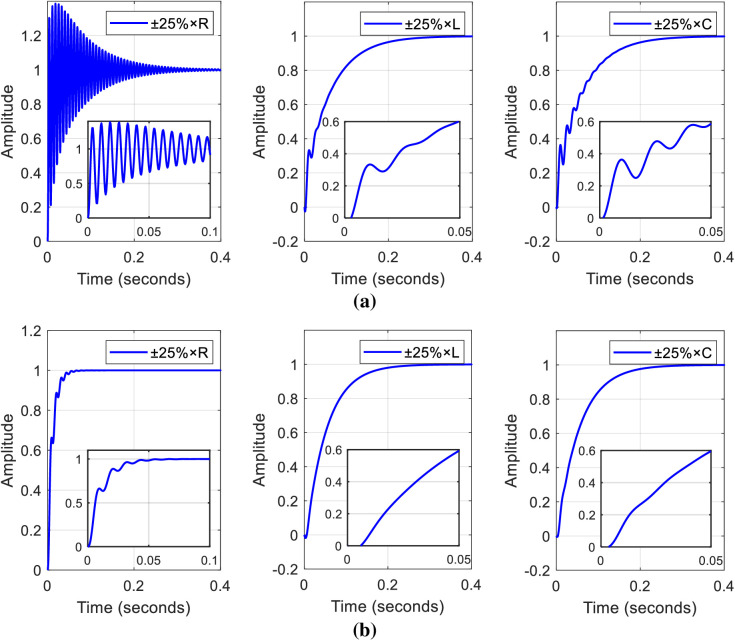
Effects of ±25% parameter variations on ISA-PI and single PI controllers: outcomes illustrating the impact of inductance, capacitance, and load resistance variations on a) single PI controller and b) the ISA-PI controller.

Parameter variations are critical in real-world applications, impacting system behavior due to deviations from nominal values. Designing controllers that can accommodate these variations is essential for maintaining stability and performance. Robust control techniques enable the development of controllers capable of adapting to parameter uncertainties. In the mathematical modelling process, the parasitic resistances of the inductor and capacitor were not explicitly included in the transfer function estimation. However, their impact was indirectly considered through the introduction of disturbance and noise during the controller design phase. By adding these factors, a more realistic operating environment was simulated, capturing the effect of non-idealities in the system. The current approach of incorporating disturbances and noise was deemed an effective method to account for system imperfections. In this study, a ±25% variation in inductance, capacitance, and load resistance is applied to the transfer function of the proposed system. The simulation has been conducted for multiple values of each parameter, with 10 samples, and a time vector spanning 1000 points with adjusted spacing of 0.0005 between each point. Fig 9a, b show the outcomes for an ISA-PI controller and a single PI controller, respectively.

In the proposed system, the duty cycle is feedback-controlled through a combination of prelookup and one-dimensional lookup table structures to facilitate adaptive behaviour, thereby optimizing system performance without the necessity for extensive manual tuning. The prelookup table effectively identifies the position of the duty cycle input within a defined set of intervals, referred to as “Breakpoint data.” Specifically, this table generates an index k and a fraction f, wherek denotes the interval containing the input u, andf indicates the normalized position of the input within that interval, constrained by the range 0.2 ≤ f ≤ 0.8. This methodology enables real-time adaptation of the duty cycle, which is limited to the range of 0.2 to 0.8.

The one-dimensional lookup table conducts interpolated lookups based on the input’s position relative to the predefined breakpoints. In this study, 20 breakpoints were selected, spanning from 0.01 to 0.9. This configuration allows for effective adaptation within a critical operating range, facilitating a finely tuned response from the controller while significantly reducing the need for manual adjustments.

The selection of breakpoint values, ranging from 0.1 to 0.9, alongside the prelookup parameters of 0.2≤f≤0.8, was grounded in a thorough analysis of empirical evidence, performance optimization, and simulation results. This selection process considered the presence of output noise quantified at $1\times 10^{-14}$ with a sample time of 0.01, utilizing a band-limited white noise block. Furthermore, the system was designed to account for disturbances arising from a PWM signal, also quantified at a value of 0.01, implemented through a step block.

By employing this adaptive structure, the system achieves automatic adaptation, ensuring robust performance across various applications. This adaptive mechanism is essential for minimizing tuning complexity while simultaneously maintaining system stability and efficiency.

**Fig 10:**
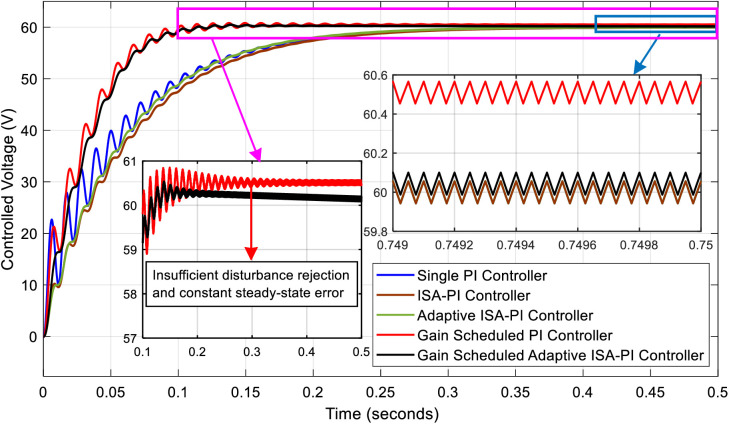
Comparative analysis of control strategies under noise and disturbance: evaluation of the proposed controller against four alternative strategies in the presence of intrinsic system variances.

The system can efficiently manage these variations by incorporating strong control techniques that ensure stable operation and good performance over a range of settings. In addition, a comparative analysis of four alternative control strategies, with the addition of a new feature at a later stage, is part of the performance evaluation of the proposed controller. This evaluation, shown in Fig 10, is carried out in the presence of noise and disturbances – two phenomena that are intrinsic and unavoidable in control system applications, especially in the case of non-linear plant dynamics.

**Fig 11:**
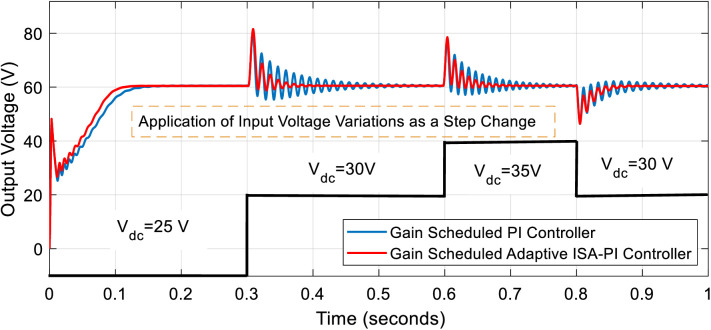
Output voltage response to input voltage variations using gain scheduled PI and gain scheduled adaptive ISA-PI Controllers.

To observe the effects of input voltage variations along with noise and disturbances on the system, step changes were applied to the input voltage. Specifically, from 0 to 0.3 seconds, the input voltage was set to 25 V. It was then increased to 30 V between 0.3 and 0.6 seconds, further raised to 35 V from 0.6 to 0.8 seconds, and finally returned to the nominal value of 30 V between 0.8 and 1 second. The results for both the gain scheduled PI controller and the proposed gain scheduled adaptive ISA-PI controller are presented in Fig 11.

For the initial 0.3-second interval, the rise time using the gain scheduled PI controller was measured as 92.876 ms, whereas the proposed gain-scheduled adaptive ISA-PI controller achieved a reduced rise time of 78.757 ms. The settling time for the first controller was recorded as 121.527 ms, while the proposed controller demonstrated an improved settling time of 98.274 ms. Notably, no overshoot was observed in this phase for either controller.

A more detailed comparison was conducted during the 0.3 to 0.6-second interval, as this period exhibited the most significant variations. The maximum percentage overshoot for the gain scheduled PI controller was recorded as 19.67%, whereas for the proposed controller, it was slightly higher at 21.26%. However, for the second and third peak values, the gain scheduled PI controller exhibited overshoots of 12.40% and 9.552%, respectively, while the proposed control approach resulted in significantly lower values of 8.415% and 4.094%. Additionally, while the system under the gain-scheduled PI controller reached a steady-state in 224 ms during this interval, the proposed controller achieved steady-state in just 50 ms, highlighting its superior transient response.

**Fig 12:**
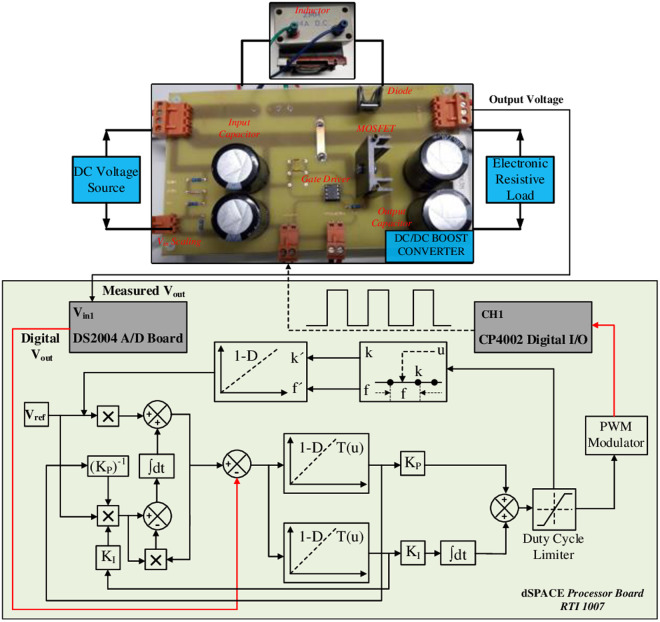
Experimental setup for assessing the proposed approach.

The experimental setting designed to assess the effectiveness of the suggested approach is shown in Fig 12. Important components of this setup include the DC-DC boost converter, an electronic resistive load that allows for constant DC voltage output, a DC voltage source, and a dSPACE real-time interface (RTI) hardware-in-the-loop (HIL) control system. The RTI setup consists of the RTI 1007 CPU board, the DS2004 high-speed A/D converter, and the CP4002 timing and digital I/O boards.

**Fig 13:**
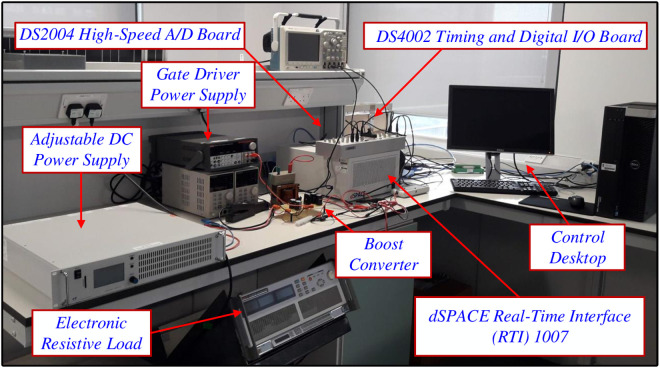
Test bench configuration of the custom-designed boost converter.

The test bench configuration for the experiment is illustrated in Fig 13. The primary DC input source for the custom-designed boost converter is a programmable 4 kW DC power supply (LAB/SMS-4600), capable of delivering a maximum output voltage of 600 V and a maximum output current of 7 A. Additionally, an adjustable bench power supply with three outputs (AL991A-48 W power rating) is utilized to energize the converter, offering a voltage range from −15 V to +15 V. For applying the desired output load resistance, a high-performance DC electronic load (3362F high voltage DC electronic load 500V, 60A, 1800W) is employed.

**Fig 14:**
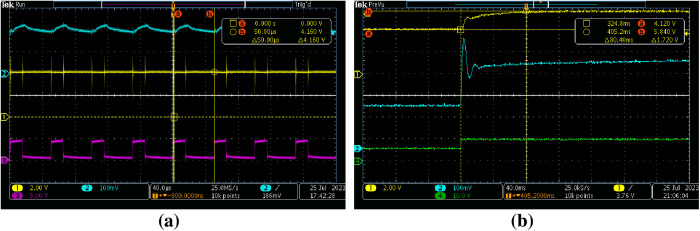
Open-loop system response at 20 kHz switching frequency a) 0.3 duty cycle, b) duty cycle change from 0.3 to 0.5.

Fig 14a shows the open-loop system operating with a 0.3 duty cycle and a 20 kHz switching frequency. Fig 14b depicts the transient response of the open-loop system when the duty cycle is altered from 0.3 to 0.5 while maintaining a switching frequency of 20 kHz.

**Fig 15:**
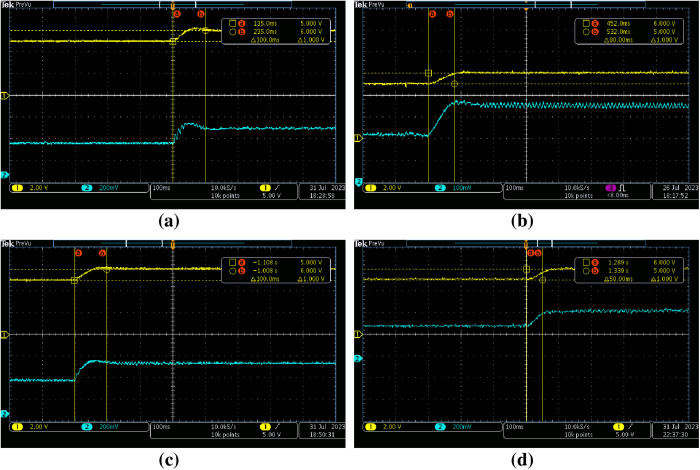
Transient response of proposed DCBC under various control strategies a) single PI, b) cascade PI, c) ISA-PI with constant set-point weight, d) adaptive gain scheduled ISA-PI.

Fig 15a–d demonstrate the transient response of the proposed DCBC under various control strategies, encompassing single PI, cascade PI, ISA-PI with a constant set-point weight $d^{m}$, and adaptive gain scheduled ISA-PI controllers, respectively. These figures provide insights into the dynamic behavior of the system across different control schemes, offering valuable comparisons for performance evaluation.

The simulation results shown in Fig 10 are in accordance with the experimental results. Fig 15a shows underdamped behaviour resulting from intrinsic characteristics. This is caused by the energy storage components, the capacitor and inductor, and single PI controller was insufficient to remove it. Although this issue was eliminated with the use of cascade PI and ISA-PI (with constant set-point weight) techniques, an overshoot has emerged, albeit sightly. The proposed adaptive gain scheduled ISA-PI controller exhibits noticeable improvements in step response characteristics. Although there was slight overshoot in the other methods, the proposed control scheme totally eliminated this and reduced the steady-state time by half.

The improved transient response demonstrates the proposed controller’s effectiveness in mitigating intrinsic system challenges, such as the interaction between energy storage components and switching dynamics. Overall, these findings highlight the importance of advanced control strategies in addressing inherent limitations and improving system performance and robustness

## 6. Conclusion and future work

This paper introduces a novel and sophisticated control scheme for a DCBC through the implementation of an adaptive gain scheduled ISA-PI controller. This controller effectively addresses the inherent non-minimum phase behavior associated with a right-half plane zero and navigates the complexities of nonlinear dynamics during CCM. By incorporating a distinct adjustable parameter within the controller structure, the proposed approach enhances adaptability to varied operating conditions.

The Adaptive ISA-PI controller integrates the real-time duty cycle value, providing a precise alternative to conventional tuning variables. This single controllable parameter can be dynamically adjusted using a meticulously designed look-up table based on the loop-shaping technique. The efficacy of the proposed control system is validated through thorough verification using MATLAB/Simulink, including comprehensive comparison assessments against single PI, cascade PI, and ISA-PI controllers with constant set-points. The evaluation primarily focuses on the system’s accuracy in tracking required signals and efficiently controlling plant process variables while minimizing delays and overshoot.

Furthermore, the research findings are corroborated by experimental validation conducted on a dSPACE RTI 1007 CPU, along with DS2004 high-speed A/D and CP4002 timing and digital I/O boards. The testing results highlight the superior performance of the introduced adaptive gain scheduled ISA-PI controller, showcasing a twofold improvement in tracking speed and significantly enhanced disturbance rejection.

Future studies could explore the extension of this control scheme to other power electronic systems and examine its resilience to disturbances and uncertainties in practical settings. Additionally, further improvements may involve the development of advanced tuning techniques aimed at maximizing the controller’s performance across various operating environments, thereby enhancing its applicability and effectiveness in real-world scenarios.

## References

[1] Subhani N, May Z, Alam MDK, Khan I, Hossain MDA, Mamun S. An improved non-isolated quadratic DC-DC boost converter with ultra high gain ability. IEEE Access. 2023;11:11350–63. doi: 10.1109/access.2023.3241863

[2] Marahatta A, Rajbhandari Y, Shrestha A, Phuyal S, Thapa A, Korba P. Model predictive control of DC/DC boost converter with reinforcement learning. Heliyon. 2022;8(11):e11416. doi: 10.1016/j.heliyon.2022.e11416 36387550 PMC9650005

[3] Cheng X-F, Liu C, Wang D, Zhang Y. State-of-the-art review on soft-switching technologies for non-isolated DC-DC converters. IEEE Access. 2021;9:119235–49. doi: 10.1109/access.2021.3107861

[4] Kart S, Demir F, Kocaarslan İ, Genc N. Increasing PEM fuel cell performance via fuzzy-logic controlled cascaded DC-DC boost converter. Int J Hydrogen Energy. 2024;54:84–95. doi: 10.1016/j.ijhydene.2023.05.130

[5] Subhani N, May Z, Alam MK, Mamun S. An enhanced gain non-isolated quadratic boost DC-DC converter with continuous source current. PLoS One. 2023;18(12):e0293097. doi: 10.1371/journal.pone.0293097 38060480 PMC10703286

[6] Anshory I, Jamaaluddin J, Wisaksono A, Sulistiyowati I, Rintyarna BS, et al. Optimization DC-DC boost converter of BLDC motor drive by solar panel using PID and firefly algorithm. Results Eng. 2024;21:101727. doi: 10.1016/j.rineng.2023.101727

[7] Daraz A, Basit A, Zhang G. Performance analysis of PID controller and fuzzy logic controller for DC-DC boost converter. PLoS One. 2023;18(10):e0281122. doi: 10.1371/journal.pone.0281122 37856453 PMC10586690

[8] Goyal VK, Shukla A. Isolated DC–DC boost converter for wide input voltage range and wide load range applications. IEEE Trans Ind Electron. 2021;68(10):9527–39. doi: 10.1109/tie.2020.3029479

[9] Guo Q, Bahri I, Diallo D, Berthelot E. Model predictive control and linear control of DC–DC boost converter in low voltage DC microgrid: an experimental comparative study. Control Eng Pract. 2023;131:105387. doi: 10.1016/j.conengprac.2022.105387

[10] Vyapari S, Viju Nair. R. A Comprehensive Analysis on the Effect of Right Half Plane Zero in the Control of Power Converters. In: 2022 IEEE International Conference on Industrial Technology (ICIT). 2022: 1–5. doi: 10.1109/icit48603.2022.10002804

[11] Goudarzian A, Khosravi A, Raeisi HA. Analysis of a step-up dc/dc converter with capability of right-half plane zero cancellation. Renew Energy. 2020;157:1156–70. doi: 10.1016/j.renene.2020.05.088

[12] Goudarzian A, Khosravi A, Raeisi HA. Modeling and implementation of a new boost converter with elimination of right‐half ‐plan zero. Int Trans Electr Energ Syst. 2020;30(9). doi: 10.1002/2050-7038.12476

[13] Goudarzian A. A new technique for right half plane zero elimination from dynamics of a boost converter using magnetic coupling concept. CW. 2021;49(3):281–93. doi: 10.1108/cw-01-2021-0013

[14] Kumar P, Ajmeri M. Comparison of control techniques for a DC-DC boost converter exhibiting non-minimum phase behaviour. In: 2022 2nd International Conference on Emerging Frontiers in Electrical and Electronic Technologies (ICEFEET), 2022. 1–6. doi: 10.1109/icefeet51821.2022.9848100

[15] Chincholkar SH, Malge SV, Patil SL. Design and analysis of a voltage-mode non-linear control of a non-minimum-phase positive output elementary luo converter. Electronics. 2022;11(2):207. doi: 10.3390/electronics11020207

[16] Yuan X, Wang K, Yang Z, Cao H. A novel modulation for four‐switch Buck‐boost converter to eliminate the right half plane zero point. Adv Control Appl. 2024;6(3). doi: 10.1002/adc2.223

[17] Abbas G, Samad MA, Gu J, Asad MU, Farooq U. Set-point tracking of a DC-DC boost converter through optimized PID controllers. In: 2016 IEEE Canadian Conference on Electrical and Computer Engineering (CCECE). 2016: 1–5. doi: 10.1109/ccece.2016.7726841

[18] Abbas G, Farooq U, Gu J, Asad MU. Controller design for low-input voltage switching converters having non-minimum phase characteristics. In: 2015 IEEE 28th Canadian Conference on Electrical and Computer Engineering (CCECE). 2015: 1294–8. doi: 10.1109/ccece.2015.7129465

[19] Wu Y, Cheng X, Yang H, Zong Z, Wei Y. Research on DC-DC converter based on right half plane zero point elimination. In: 2020 IEEE 9th Joint International Information Technology and Artificial Intelligence Conference (ITAIC). 2020: 937–41. doi: 10.1109/itaic49862.2020.9338934

[20] Pan C, Zhan C, Martins RP, Lam C-S. A continuous-output-current buck-boost converter without Right-Half-Plane-Zero (RHPZ). IEEE Trans Circuits Syst I. 2023;70(12):4719–28. doi: 10.1109/tcsi.2023.3308781

[21] Goudarzian A, Mirzaeian Dehkordi B, Abjadi N, Adib E. Design of a switched‐capacitor boost converter utilizing magnetic coupling with capability of right‐half plane zero elimination. IET Power Electron. 2020;14(1):211–24. doi: 10.1049/pel2.12026

[22] Salehi SM, Yazdian A. A step-up DC-DC converter with high voltage gain and eliminated right half plane zero. In: 2023 14th Power Electronics, Drive Systems, and Technologies Conference (PEDSTC). 2023: 1–6. doi: 10.1109/pedstc57673.2023.10087149

[23] Kumar N, Veerachary M. Stability region based robust controller design for high-gain boost DC–DC converter. IEEE Trans Ind Electron. 2021;68(3):2246–56. doi: 10.1109/tie.2020.2972448

[24] Hajihosseini M, Andalibi M, Gheisarnejad M, Farsizadeh H, Khooban M-H. DC/DC power converter control-based deep machine learning techniques: real-time implementation. IEEE Trans Power Electron. 2020;35(10):9971–7. doi: 10.1109/tpel.2020.2977765

[25] Qi W, Zong G, Zheng WX. Adaptive event-triggered SMC for stochastic switching systems with semi-markov process and application to boost converter circuit model. IEEE Trans Circuits Syst I. 2021;68(2):786–96. doi: 10.1109/tcsi.2020.3036847

[26] Hassan M, Su C-L, Chen F-Z, Lo K-Y. Adaptive passivity-based control of a DC–DC boost power converter supplying constant power and constant voltage loads. IEEE Trans Ind Electron. 2022;69(6):6204–14. doi: 10.1109/tie.2021.3086723

[27] Irshad M, Vemula NK, Devarapalli R, Kumar GVN, Knypiński Ł. An optimized integral performance criterion based commercial PID controller design for boost converter. J Electr Eng. 2024;75(4):258–67. doi: 10.2478/jee-2024-0032

[28] Birs I, Muresan C, Nascu I, De Keyser R. Experimental comparison between discrete time and event-based PID controllers on a nonlinear process. In: 2021 International Conference on Electrical, Computer, Communications and Mechatronics Engineering (ICECCME). 2021: 1–6. doi: 10.1109/iceccme52200.2021.9590879

[29] Balamurugan S, Umarani A. Study of discrete PID controller for DC motor speed control using MATLAB. In: 2020 International Conference on Computing and Information Technology (ICCIT-1441). 2020: 1–6. doi: 10.1109/iccit-144147971.2020.9213780

[30] Lopez-Sanchez I, Moreno-Valenzuela J. PID control of quadrotor UAVs: a survey. Ann Rev Control. 2023;56:100900. doi: 10.1016/j.arcontrol.2023.100900

[31] Mien TL, An VV, Tam BT. A Fuzzy-PID controller combined with PSO algorithm for the resistance furnace. Adv Sci Technol Eng Syst J. 2020;5(3):568–75. doi: 10.25046/aj050371

[32] Aboelhassan A, Abdelgeliel M, Zakzouk EE, Galea M. Design and implementation of model predictive control based PID controller for industrial applications. Energies. 2020;13(24):6594. doi: 10.3390/en13246594

[33] Della Santina C, Duriez C, Rus D. Model-based control of soft robots: a survey of the state of the art and open challenges. IEEE Control Syst. 2023;43(3):30–65. doi: 10.1109/mcs.2023.3253419

[34] Irmak E, Güler N. A model predictive control-based hybrid MPPT method for boost converters. Int J Electron. 2019;107(1):1–16. doi: 10.1080/00207217.2019.1582715

[35] Gheisarnejad M, Mohammadzadeh A, Khooban M-H. Model predictive control based Type-3 fuzzy estimator for voltage stabilization of DC power converters. IEEE Trans Ind Electron. 2022;69(12):13849–58. doi: 10.1109/tie.2021.3134052

[36] Gholamzadehmir M, Del Pero C, Buffa S, Fedrizzi R, Aste N. Adaptive-predictive control strategy for HVAC systems in smart buildings – A review. Sustain Cities Soc. 2020;63:102480. doi: 10.1016/j.scs.2020.102480

[37] Noye S, Mulero Martinez R, Carnieletto L, De Carli M, Castelruiz Aguirre A. A review of advanced ground source heat pump control: Artificial intelligence for autonomous and adaptive control. Renew Sustain Energy Rev. 2022;153:111685. doi: 10.1016/j.rser.2021.111685

[38] Mumtaz F, Zaihar Yahaya N, Tanzim Meraj S, Singh B, Kannan R, Ibrahim O. Review on non-isolated DC-DC converters and their control techniques for renewable energy applications. Ain Shams Eng J. 2021;12(4):3747–63. doi: 10.1016/j.asej.2021.03.022

[39] Chandrasekar B, Nallaperumal C, Padmanaban S, Bhaskar MS, Holm-Nielsen JB, Leonowicz Z, et al. Non-isolated high-gain triple port DC–DC buck-boost converter with positive output voltage for photovoltaic applications. IEEE Access. 2020;8:113649–66. doi: 10.1109/access.2020.3003192

[40] Ping X, Hu J, Lin T, Ding B, Wang P, Li Z. A survey of output feedback robust MPC for linear parameter varying systems. IEEE/CAA J Autom Sinica. 2022;9(10):1717–51. doi: 10.1109/jas.2022.105605

[41] Hemelhof L, Markovsky I, Patrinos P. Data-driven output matching of output-generalized bilinear and linear parameter-varying systems. In: 2023 European Control Conference (ECC). 2023: 1–6. doi: 10.23919/ecc57647.2023.10178404

[42] Lamouchi R, Raissi T, Amairi M, Aoun M. On interval observer design for active Fault Tolerant Control of Linear Parameter-Varying systems. Syst Control Lett. 2022;164:105218. doi: 10.1016/j.sysconle.2022.105218

